# Lateralization of retinal structure correlated with behavioral lateralization in the oval squid *Sepioteuthis lessoniana*

**DOI:** 10.1186/s40851-026-00268-5

**Published:** 2026-09-17

**Authors:** Yuma Sakurai, Hisanori Kohtsuka, Ryosuke Kimbara, Toru Miura, Nobuaki K. Tanaka

**Affiliations:** 1 Division of Biology, Department of Biological Sciences, School of Science, Hokkaido University, North 10, West 8, Kita-ku, Sapporo, 060-0810 Japan; 2 Misaki Marine Biological Station, School of Science, University of Tokyo, Miura, Kanagawa 238-0225 Japan; 3 Molecular Genetics Unit, Okinawa Institute of Science and Technology, Onna, Okinawa 904-0495 Japan; 4 Graduate School of Life Science, Hokkaido University, North 10, West 8, Kita-ku, Sapporo, 060-0810 Japan; 5 Faculty of Science, Hokkaido University, North 10, West 8, Kita-ku, Sapporo, 060-0810 Japan; 6 Present address: Research and Education Center for Natural Sciences, Keio University, 4-1-1, Hiyoshi, Kohoku, Yokohama 223-8521 Japan

**Keywords:** Cephalopod, Chromocenter, FDTD simulation, Laterality, Photoreceptor cell, Retina, Visual lateralization

## Abstract

**Background:**

Cephalopods, including *Sepioteuthis lessoniana*, preferentially orient either the left or right eye toward visual targets during various visually guided behaviors, as most of their visual field is monocular. Such visual lateralization is widely observed in the animals whose eyes are positioned laterally on the head. However, the neural mechanisms underlying visual lateralization remain largely unclear. In this study, we investigated whether lateralization of retinal structures is correlated with behavioral lateralization toward conspecifics in the oval squid *S. lessoniana*. We analyzed lateralization in juvenile *S. lessoniana* reared in a container from eggs for 86 days, when they were maintained at a density of 0.15 individuals/L and exhibited a normal schooling behavior, one of the social behaviors in this species. Visual lateralization was quantified by analyzing which eye animals preferentially faced toward conspecifics and by calculating its laterality index. Lateralization of each retina was assessed by measuring focal length, photoreceptor cell density in the posterior retina, and the proportion of photoreceptor cells containing a single chromocenter in their nuclei. The optical properties of the eye were also examined by testing whether light passing through the retina was reflected by the argentea, the outer reflective layer of the eye. In addition, three-dimensional finite-difference time-domain simulations were performed to investigate how the number of chromocenters affects light collection in photoreceptor cells.

**Results:**

Among the retinal parameters examined, only the lateralization of the proportion of photoreceptor cells containing a single chromocenter showed a significant correlation with visual lateralization toward conspecifics. Optical experiments demonstrated that light passing through the retina was reflected from the inner surface of the argentea. Simulations further indicated that photoreceptor cells containing a single chromocenter collect reflected light onto the outer segment more efficiently than those containing multiple chromocenters.

**Conclusions:**

These findings suggest that the dominant eye in *S. lessoniana* may achieve higher contrast sensitivity because it contains a higher proportion of photoreceptor cells containing a single chromocenter, which more efficiently collects light on the outer segment. Such lateralization in retinal structure may enhance the detection of weak visual signals from conspecifics and contribute to visual lateralization during social interactions.

**Supplementary Information:**

The online version contains supplementary material available at https://doi.org/10.1186/s40851-026-00268-5.

## Background

Cephalopods exhibit various behaviors such as preying, camouflage, and communication using their body patterns [1]. These behaviors rely on their unique visual system. Although they share lens eyes with vertebrates, their retinal organization differs [2]. The cephalopod retina contains only a single type of neuron, the photoreceptor cells [3]. In cephalopods, the postsynaptic neurons to photoreceptor cells are located in the primary visual center, called the optic lobe, rather than in the retina [4, 5], unlike in the vertebrate retina, where various interneurons form a synaptic network. The photoreceptor cells in the cephalopod retina are oriented in the opposite direction to those in vertebrates. The outer segments of the photoreceptor cells are oriented toward the lens, indicating that light passing through the lens is directly absorbed by the outer segments, unlike in vertebrates. Moreover, the outer segment consists of two rhabdomeres [3], where microvilli are arranged at a consistent angle, allowing the detection of polarized light [6]. Whereas the foveal structure, where the density of cone cells is high in the vertebrate retina, has not been found in the cephalopod retina [7], the photoreceptor cells are unevenly distributed across the cephalopod retina. For example, coastal (*Euprymna morsei* and *Sepioteuthis lessoniana*) and oceanic (*Todarodes pacificus*, *Eucleoteuthis luminosa*, and *Thysanoteuthis rhombus*) squid species exhibit the highest cell densities in the dorsal/posterior and ventral/posterior retinal areas, respectively [8]. These species-specific distributions may indicate differences in posture and position when they orient toward prey and conspecifics [8].

Most visual fields in cephalopods are dominated by monocular vision [9], as their eyes are located on the sides of the head. *Sepioteuthis lessoniana*, *Octopus vulgaris*, *Sepia officinalis*, and *Sepia apama* preferentially orient their left or right eye toward visual targets such as prey, predators, and conspecifics [9–12]. *Sepia officinalis* adjusts its body color based on the brightness of the substrate, as perceived preferentially by the right eye [13]. Such visual lateralization is commonly observed across a wide range of animal taxa, including both vertebrates and invertebrates [14, 15]. Visual lateralization may reduce redundancy in visual information processing between the left and right nervous systems, thereby reducing the time required for decision making [14]. Furthermore, theoretical models suggested that alignment of lateralization across individuals can facilitate coordinated social interactions between conspecifics [16], such as aggression, affiliation, and mating [17–21], resulting in the population-level lateralization, in which most individuals share the same directional bias as an evolutionarily stable strategy [22, 23]. Whereas behavioral lateralization has been revealed in both vertebrates and invertebrates [14, 15], only a limited number of studies have revealed correlations between lateralization of neural properties and visual lateralization. In fish, *Geophagus brasiliensis* predominantly turns left or right in a T-maze, which correlates with volume differences between the left and right habenula [24]. In cephalopods, *S. officinalis* predominantly turns left in a T-maze, which correlates with volume differences between the left and right vertical and optic lobes [25].

The oval squid, *S. lessoniana*, is an active visual predator along coastal areas during both day and night [26–29]. Schooling behavior, one of the social behaviors of this species, starts around 60 days after hatching [30]. Given that social interactions rely on visual signals from conspecifics, visual lateralization may play a key role in processing these signals. Indeed, *S. lessoniana* older than 60 days preferentially orients either the left or right eye toward conspecifics [12], this lateralization correlates with the position of each animal within the school [31]. It has been reported that the visual lateralization is not correlated with lateralization of optic lobe volumes, indicating that other neural properties may cause visual lateralization [12].

In this study, to reveal lateralization in the eye structures underlying visual lateralization in *S. lessoniana*, we investigated the correlation between visual lateralization and the lateralization of focal length and retinal organization, such as the density of photoreceptor cells and the proportion of photoreceptor cells containing a single chromocenter within their nuclei. In mammalian rod cells, nuclei are partially occupied by chromocenters, which are clusters of heterochromatin in which DNA is tightly packed [32]. The number of chromocenters in mammalian rod cells is correlated with lifestyle, specifically whether the animal is diurnal or nocturnal; nocturnal animals have a single chromocenter in rod cells, whereas diurnal animals have multiple chromocenters [32]. A simulation study indicated that rod cells containing a single chromocenter focus light on the outer segment more efficiently than those containing multiple chromocenters [32]. Additionally, mice with rod cells containing a single chromocenter exhibited higher contrast sensitivity than those with multiple chromocenters, although both groups showed similar visual acuity [33]. We also investigated how heterochromatin in photoreceptor cells contributes to light reception in the dominant eye of *S. lessoniana.*

## Methods

### Sampling and rearing animals

We used *S. lessoniana*, referred to as “Shiro-ika” in Japanese (*Sepioteuthis* sp. 2 in [34]). Egg cases of *S. lessoniana* were collected from the coast of Uruma, Japan, in cooperation with the University of the Ryukyus and at the Misaki Marine Biological Station of the University of Tokyo (Miura, Japan), or were kindly provided by Ushimado Marine Institute of Okayama University (Setouchi, Japan), Shimoda Marine Institute of the University of Tsukuba (Shimoda, Japan), Blue Corner (Numazu, Japan), and Yoka Yoka Diving Service (Amakusa, Japan). The eggs were transferred to artificial seawater (TetraMarin Salt Pro, Tetra, Melle, Germany) in a circular tank (1,360 mm in diameter, 1,100 L in volume; SAKAS Inc., Hiroshima, Japan) at the Genome Dynamics Center of Hokkaido University. The tank was equipped with a closed seawater system containing a temperature controller, ultraviolet sterilizer (Aqua Inc., Tokyo, Japan), and protein skimmer (EV-2000, AquaC, South Carolina, USA). Water temperature, salinity, pH, and dissolved oxygen concentration were maintained at 24.5–25.0 °C, 32–34 practical salinity units (PSU), ˃7.8, and ˃6.8 mg/L, respectively. Ammonia and nitrite concentrations were measured using the Sera NH_4_/NH_3_ test (Sera, Heinsberg, Germany) and Tetra NO_2_^−^ test (Tetra) and were maintained at 0 and <0.3 mg/L, respectively. The light:dark (LD) cycle was not controlled artificially.

The hatching date for each population was approximated as the day on which the highest number of *S. lessoniana* hatched. Hatchlings were collected daily in smaller containers (400 mm in diameter, 495 mm in depth, and 70 L in volume) floating within the tank. The animals were reared in the containers from the hatchling to juvenile stage. The bottom of each container was opened and covered with a net to allow seawater exchange with the tank. The containers were enriched with artificial seaweed placed on the net. The densities of hatchlings and juveniles (80 days old) in each container were set at ˂7.14 and <0.15 animals/L, respectively. Hatchlings and juveniles were fed live adult mysids, *Neomysis japonica,* and frozen anchovies, *Engraulis japonica*, respectively, three times a day until all animals stopped hunting. Dead animals, feces, and remaining prey were removed from the tank immediately after feeding. We did not observe any abnormal behaviors in the containers during rearing.

All animal experiments were approved by Hokkaido University and were performed according to the guidelines of the university and the rules generally applied in EU countries [35, 36].

### Behavioral recording

Five juvenile *S. lessoniana* aged 78–86 days were used (mantle length: 39.2–75.5 mm), which exhibited schooling behavior within the container. Behavioral experiments were performed as described by Sakurai and Ikeda [12]. Briefly, 10 days before the behavioral experiments, juveniles were anesthetized in 0.8% ethanol in artificial seawater at 25 °C. After confirming anesthesia according to the assessment of anesthesia in cephalopods described by Fiorito et al. [35], we marked them with visible implant fluorescent elastomer (VIE) tags (Northwest Marine Technology, Shaw Island, WA, USA) to visually distinguish individual animals [37]. During anesthesia, we did not observe any typical adverse response patterns [38]. The behavioral experiments were conducted in a rectangular tank (750 mm × 550 mm × 300 mm). The tank was divided into smaller and larger compartments by a double-layered barrier consisting of transparent glass and a black styrene plate. The smaller compartment occupied one-third of the tank. One subject was placed in the smaller compartment and the others in the larger compartment for 1 h to acclimate to the tank. The black plate was then removed to present the conspecifics for 10 min. The animals were video recorded at 30 Hz using a digital camera (FDR-AX60; Sony, Tokyo, Japan) placed above the tank. During the experiments, we did not observe stress responses such as inking or jetting; instead, the animals displayed normal swimming behavior.

### Histology

Following the behavioral experiments, each animal was euthanized in 2% ethanol in artificial seawater at 25 °C [35], and its mantle length was measured. While the animals were immersed in the euthanasia solution, they initially exhibited normal swimming behavior, then showed a gradual decrease in breathing and arm and fin movements. Death was determined by confirming cardiac arrest according to the guidelines of Fiorito et al. [35]. The head was fixed in 4% paraformaldehyde (PFA) in phosphate-buffered saline (PBS) for 48 h at 25 °C with shaking and then stored at 4 °C in a refrigerator until further dissection.

After washing the head with PBS, both eyes were excised and their focal lengths were measured. The focal length was defined as the distance between the center of the lens and the medial surface of the eye. The iris, lens, and argentea were removed from the eye, and the retina was flattened by cutting it from the periphery toward the center. A square piece (approximately 5 mm × 5 mm) from the posterior area, where the highest cell density was observed in the retina of *S. lessoniana* [8], was embedded in 25% albumin/4.8% gelatin in distilled water, fixed in 8% PFA/PBS for 3 days, and sliced using the LinearSlicer PRO 7 (Dosaka EM, Kyoto, Japan) at a thickness of 150 μm [39]. The slices were shaken in 1 μg/mL propidium iodide (Dojindo Laboratories, Kumamoto, Japan) or 10 μg/mL Hoechst 33342 (Dojindo Molecular Technologies, Kumamoto, Japan) in PBS containing 2% Triton X-100, washed with PBS, and then mounted in 80% glycerol/PBS.

### Reconstruction and analysis of confocal images

Confocal serial optical images (160 µm × 160 µm) of the photoreceptor cell layer were captured at 0.31–1.0 μm z-intervals using a laser scanning confocal microscope (LSM700 or LSM980, Carl Zeiss, Oberkochen, Germany) equipped with a C-Apochromat 40× objective. We counted the number of photoreceptor cells included in the photoreceptor cell layer within a 0.1 mm × 0.1 mm area of the confocal images and considered the sum as the density of photoreceptor cells.

Three-dimensional reconstruction of the confocal images was performed using ZEN 2012 (Zeiss) or ITK-SNAP (version 4.0.2) [40]. The Cell Counter plugin within Fiji 1.54f [41] (https://imagej.net/plugins/cell-counter) was used to count the number of nuclei in each image after adjusting image brightness and contrast using Fiji. The volume of the photoreceptor cell layer was estimated by multiplying the sum of the layer areas in each confocal image by the z-interval.

### Laterality index

The dominant eye in each animal was determined as reported by Sakurai and Ikeda [12]. Briefly, the visual field of each eye was considered as the space between two lines, each of which passes through the lens and the anterior or posterior end of the retina. The overlapping areas between the visual fields of the left and right eyes were regarded as binocular visual fields, whereas the visual fields of the left and right eyes from which the binocular visual field was subtracted were defined as the left and right monocular visual fields, respectively. The number of conspecifics was counted in each visual field in 10 images (one image per minute from the movie recorded for 10 min). The laterality index of visual lateralization (*LI*_*V*_) was calculated for each animal using the following formula:

$$L{I_V} = {{RV - LV} \over {RV + LV + BV}}$$

where *RV*, *LV*, and *BV* are the numbers of images in which the right monocular, left monocular, and binocular visual fields, respectively, include the greatest number of conspecifics. *LI*_*V*_ ranges from −1 to +1, where positive values indicate that the animal dominantly faces its right eye toward conspecifics.

The laterality indices of the retinal structures such as focal length (*LI*_*F*_), the density of the photoreceptor cells (*LI*_*D*_), and the proportion of photoreceptor cells containing a single chromocenter (*LI*_*C*_) for each animal were also calculated according to the formula commonly used in studies of lateralization in animals [42]:

$$L{I_F},L{I_D},and\,L{I_C} = {{R - L} \over {R + L}}$$

where *R* and *L* represent the focal length, the cell density, and the proportion of the right and left eyes, respectively. A positive *LI* indicates that the right eye has a higher value.

### Laser application to the eyes

Three juvenile *S. lessoniana* aged 73–75 days were used (mantle length: 28.7–35.4 mm). Each animal was placed in a bucket (275 mm in diameter, 10 L in volume) surrounded by a black cloth at 25 °C for 1 hour, transferred to an opaque container (365 mm × 260 mm × 80 mm), and then anesthetized by gradually decreasing the temperature by replacing the seawater with cold seawater to minimize stress caused by anesthesia [43]. We confirmed that the behavioral responses during cold-seawater anesthesia were comparable with those observed during ethanol anesthesia. The left and right eyes were then excised under dim light conditions.

The left eye was placed over a square hole made on a stage and exposed to light from a laser pointer (TGC-006; Technologic, Tokyo, Japan, λ=532 nm) positioned over the eye at a distance of 15 cm. The light spot on the lens of the eye was 3 mm in diameter (photon flux density = 470 μmol·m^−2^·s^−1^). The light passing through the eye was reflected by a mirror placed under the hole and captured using a camera (Tough TG-6; OLYMPUS, Tokyo, Japan). Subsequently, a 3 × 3 mm square of argentea was excised from the eye, and the light passing through the eye was again captured using the camera.

For the right eye, the iris and lens were removed, and the eye was flattened by cutting it from its periphery toward the center. The argentea was then peeled off using forceps. The argentea placed on a blackboard was exposed to light from the laser pointer. The light on the black cloth, which was reflected from the inner and outer surfaces of the argentea, was captured using the camera.

The images of the transmitted and reflected light were converted into 16-bit data, and the light intensity was measured using Fiji [41]. The standard intensity was calculated from images of the light that passed through the hole in the absence of the eye and the light reflected from a mirror placed in place of the argentea. The relative brightness of the transmitted and reflected light was calculated by dividing the light intensity by the standard intensity. The photon flux density was measured using a full-spectrum quantum sensor (MQ-200; Apogee Instruments, Logan, UT, USA).

### Simulations

We performed 3D finite-difference time-domain (FDTD) simulations of light passage using libraries from the open-source FDTD package MEEP [44], based on previous simulations of light collection by the nucleus of a mouse rod cell [32]. A simulation domain was set to 30 × 100 × 30 μm with a spatial resolution of 33 pixels per μm under a refractive index of 1. Perfectly matched layers of 1 μm thickness were placed at the borders of the domain to prevent reflections from the boundaries. The sizes of the peripheral heterochromatin rims containing one, two, and three chromocenters used in the simulations were determined based on the mean lengths of each axis among 10 randomly selected photoreceptor cells (16.1, 16.9, and 16.8 µm for the longitudinal axis; 6.8, 7.1, and 7.5 µm for the major axis; and 6.0, 5.8, and 6.2 µm for the minor axis, respectively, where the major and minor axes represent the longest and shortest axes, respectively, of the tangential section of peripheral heterochromatin rims). The refractive indices of heterochromatin, euchromatin, and surrounding tissue were set to 1.415, 1.385, and 1.360, respectively [32, 45]. To simplify the simulations, other organelles were not considered in this study. The wavelength of the light was set to 500 nm, which is the peak absorbance of the photoreceptor cells in *S. lessoniana* [46]. Given that the refractive index of the surrounding tissue is 1.360, 500 nm was considered to correspond to approximately 12 pixels. The light source was placed at a distance of 40 µm from the center of the peripheral heterochromatin rims. The simulation was run for 250 periods of the light wave until the interference pattern of the electric field reached a steady state, and the electric field of each pixel was then recorded for one additional period. The recorded electric field was squared and then averaged for each pixel to obtain the mean intensity of each pixel. The normalized intensity was calculated by dividing the mean intensity of each pixel by that recorded at the light source.

### Statistical analysis

The following statistical methods were performed using RStudio (version 2024.04.0 + 735) [47]: binomial tests, simple linear regression, linear models (LMs), and F-tests to assess the statistical significance of correlations in the LMs. We also calculated effect sizes by Pearson’s correlation analysis. Data are presented as mean ± standard error of the mean. Statistical significance was set at *p* < 0.05.

## Results

We first examined the visual lateralization of five juvenile animals (78–86 days old) by analyzing which eye preferentially faced toward conspecifics within 10 min [12]. We confirmed a previous result demonstrating that each animal exhibits a preferential side for facing toward conspecifics [12], except for one animal that did not show a significant preference (*p* = 0.73, binomial test; Table 1).

**Table 1:** Summary of data for each subject of *Sepioteuthis lessoniana*

ID	ML (mm)	*LI*_*V*_ **(p)**	Focal length (mm)		**Density of photoreceptor cells (×10**^**3**^**/mm**^**3**^)		Proportion of photoreceptor cells containing a single chromocenter	
			Left	Right	Left	Right	Left	Right
1	73.1	1.0 (0.002)	8.66	8.92	816	990	0.31	0.39
2	73.4	−1.0 (0.002)	8.57	7.67	1116	1036	0.63	0.43
3	75.5	−0.9 (0.004)	8.22	7.54	1259	1018	0.45	0.36
4	72.7	0.2 (0.73)	8.36	8.51	1316	1132	0.37	0.39
5	39.2	0.7 (0.039)	5.34	4.92	1256	975	0.36	0.46

Abbreviations: ID, identification; *LI*_*V*_, laterality index of visual lateralization; ML, mantle length; *p, p* value calculated using the binomial test

### Focal length and density of photoreceptor cells of the eye were not correlated with behavioral lateralization

**Fig. 1:**
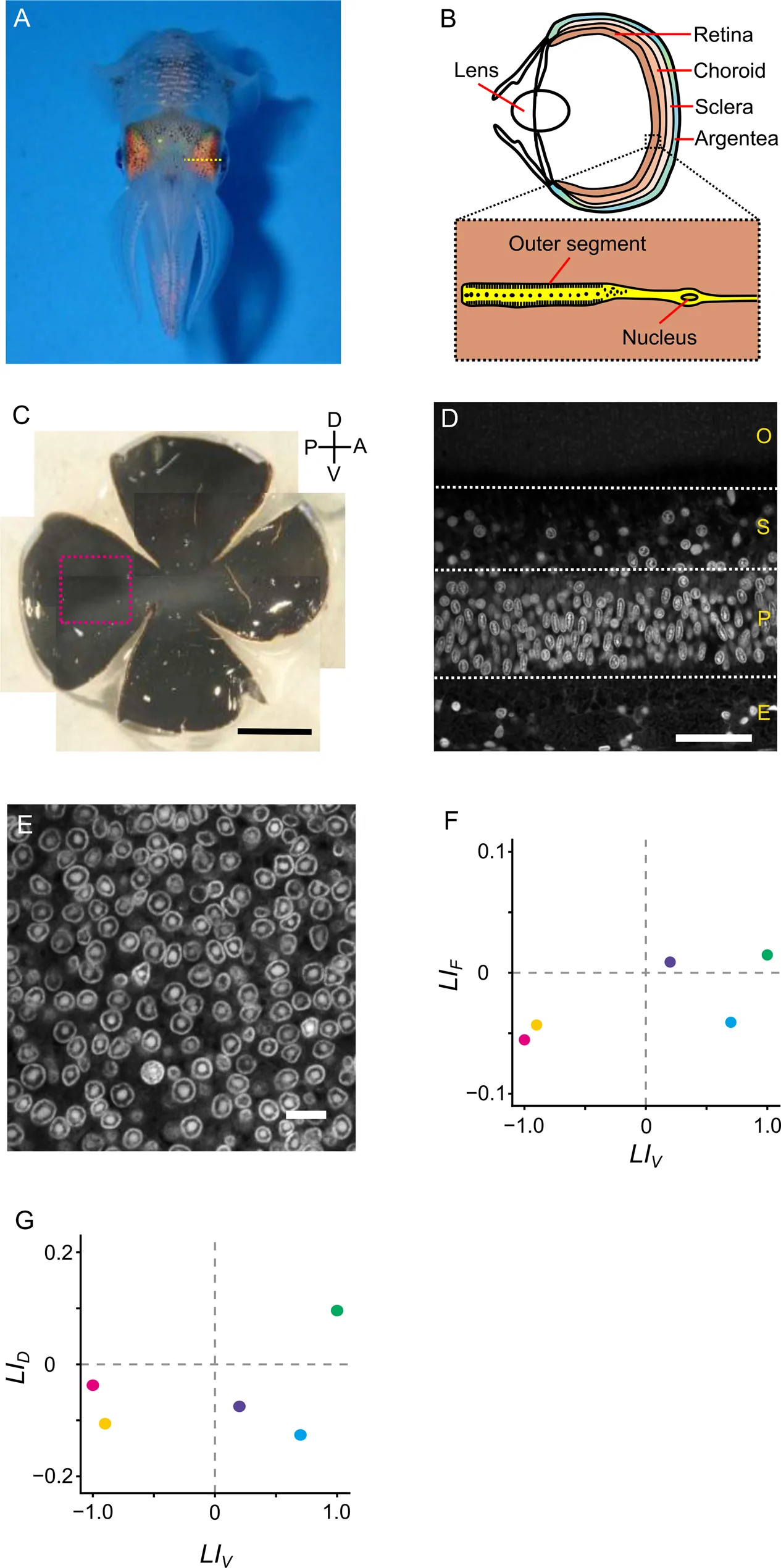
Correlation between visual lateralization and lateralization of focal length and photoreceptor cell density. (**A**) *Sepioteuthis lessoniana*. The yellow dotted line indicates the focal length measured in this study. (**B**) Schematic diagram of the eye and a photoreceptor cell in *S. lessoniana*. In cephalopods, the outer segment of the photoreceptor cell is oriented toward the lens. (**C**) Flattened retina. The magenta square indicates the posterior area where photoreceptor cells were counted in this study. (**D**) Confocal image of a transverse section of the retina stained with propidium iodide. The retina consists of four layers: the outer segment (**O**), supporting cell (**S**), photoreceptor cell (**P**), and epithelial cell (**E**) layers [3]. (**E**) Confocal image of a tangential section of the photoreceptor cell layer labeled with propidium iodide in the posterior area of the retina. Scale bars: 5 mm (**C**), 50 μm (**D**), and 10 μm (**E**). (**F**) Scatterplots of the laterality index of focal length (*LI*_*F*_) versus the laterality index of visual lateralization (*LI*_*V*_). (**G**) Scatterplots of the laterality index of photoreceptor cell density (*LI*_*D*_) versus the laterality index of visual lateralization (*LI*_*V*_). Each color in (**F, G**) represents a different animal

We examined two anatomical factors of each eye: focal length (Fig. 1A) and photoreceptor cell density in the posterior area of the retina, where the highest cell density was observed in the retinae of *S. lessoniana* (Fig. 1B–E, Table 1). We calculated the laterality index of focal length (*LI*_*F*_; see Methods) and the density of photoreceptor cells (*LI*_*D*_; see Methods) and analyzed the correlation of *LI*_*V*_ with *LI*_*F*_ and *LI*_*D*_ using LMs and Pearson’s correlation analysis. No significant correlation was observed between *LI*_*V*_ and *LI*_*F*_ (*p* = 0.20, *F*_1,3_ = 2.75, *β* = 19.5 ± 11.7, 95% CI [−3.52, 42.43], *r* = 0.69; Fig. 1F) and between *LI*_*V*_ and *LI*_*D*_ (*p* = 0.52, *F*_1,3_ = 0.54, *β* = 4.04 ± 5.52, 95% CI [−6.77, 14.86], *r* = 0.39; Fig. 1G). In summary, we did not observe a significant correlation between visual lateralization and the lateralization of focal length and cell density.

### Lateralization of the proportion of photoreceptor cells containing a single chromocenter was correlated with behavioral lateralization

**Fig. 2:**
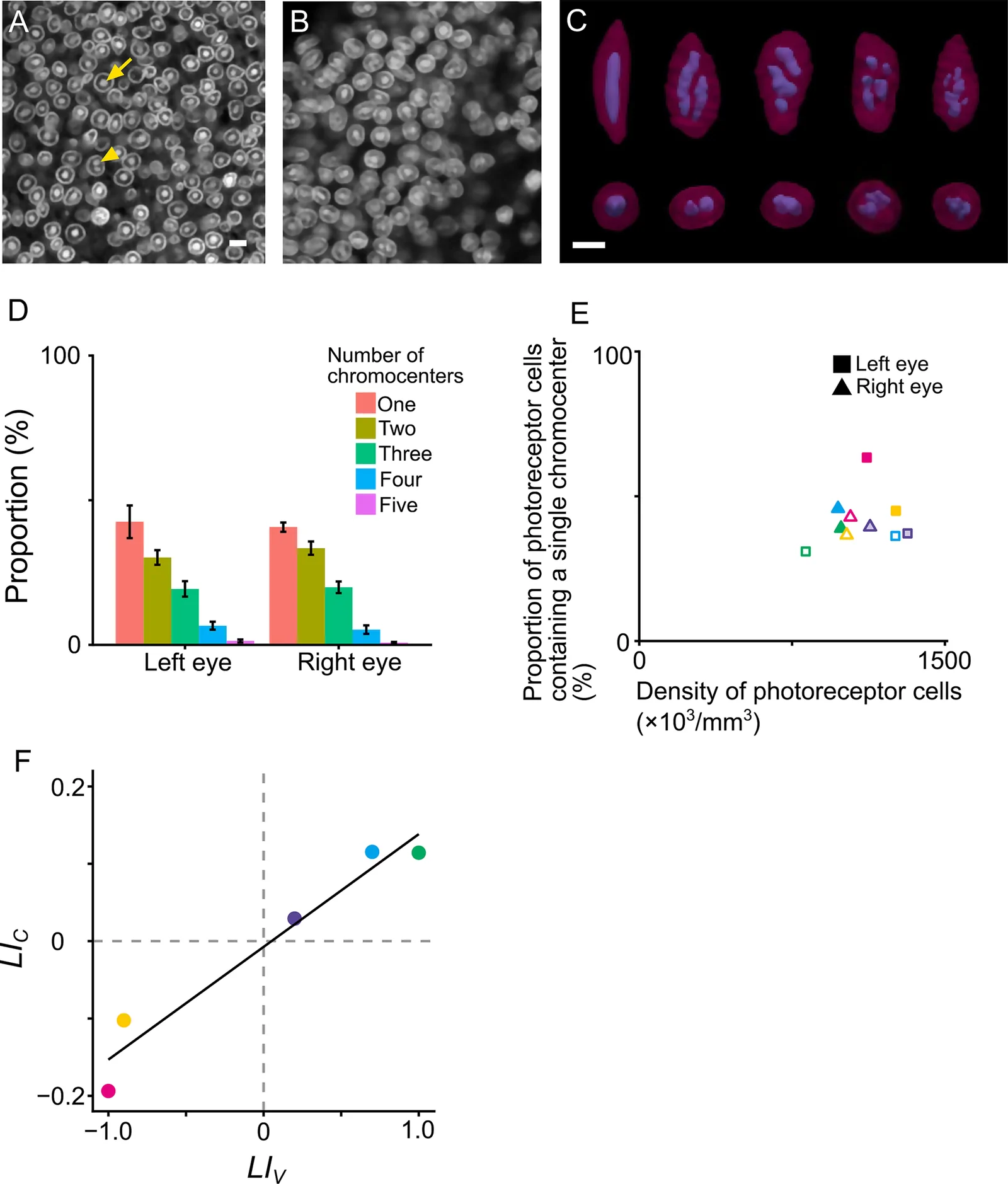
Visual lateralization correlates with lateralization of proportions of single-chromocenter photoreceptors in the retinae. (**A**) Confocal image of a tangential section of the posterior area of the retina labeled with propidium iodide. The yellow arrow and arrowhead indicate a single chromocenter and multiple chromocenters in photoreceptor cells, respectively. (**B**) Confocal image of a tangential section of the retina labeled with Hoechst 33,342. (**C**) Lateral (top) and dorsal (bottom) views of 3D-reconstructed heterochromatin structures containing one to five chromocenters (from left to right). Cyan and magenta indicate chromocenters and the peripheral heterochromatin rim, respectively. Scale bars: 5 μm (**A, C**). (**D**) Proportion of photoreceptor cells containing one to five chromocenters in the posterior area of the left and right retinae. Error bars represent the standard error of the mean. (**E**) Scatterplots of the proportion of photoreceptor cells containing a single chromocenter versus photoreceptor cell density. Filled symbols represent the dominant eye, whereas open symbols represent the non-dominant eye. Purple symbols represent a non-lateralized individual. Squares and triangles indicate the left and right eyes, respectively. (**F**) Scatterplots of the laterality index of the proportion of photoreceptor cells containing a single chromocenter (*LI*_*C*_) in the posterior area of the retina versus the laterality index of visual lateralization (*LI*_*V*_). Each color in (**E, F**) represents a different animal

Propidium iodide, which labels double-stranded nucleic acids, was used to count the number of photoreceptor cells in the retina. Propidium iodide labeled the peripheral rim along the nuclear border, including single- or multiple string-like structures within each nucleus (Fig. 2A). These structures were also labeled with Hoechst 33342 (Fig. 2B), which specifically labels double-stranded DNA, indicating that they are heterochromatin structures called chromocenters and peripheral heterochromatin rims that envelop the chromocenters [32].

The number of chromocenters within each nucleus ranged from one to five (Fig. 2C, D). We counted the chromocenters within each photoreceptor cell in the posterior area of the retina and found that most photoreceptor cells contained a single chromocenter (Fig. 2D). The proportion of photoreceptor cells containing a single chromocenter did not have a significant effect on the density of photoreceptor cells (*p* = 0.54, *F*_1,8_ = 0.40, *β* = 0.0003 ± 0.0006, 95% CI [−0.0010, 0.0018], LMs; Fig. 2E).

We then investigated whether visual lateralization (*LI*_*V*_) was correlated with the laterality index of the proportion of photoreceptor cells containing a single chromocenter (*LI*_*C*_) in the posterior area. We found that *LI*_*V*_ was positively correlated with *LI*_*C*_ (*p* = 0.004, *F*_1,3_ = 52.94, *β* = 6.49 ± 0.89, 95% CI [4.74, 8.24], LMs; *r* = 0.98, Pearson’s correlation analysis; Fig. 2F). Because rod cells containing a single chromocenter help mice exhibit higher contrast sensitivity [33], these results suggest that *S. lessoniana* orients the eye with better contrast sensitivity toward conspecifics.

Lateralization of the proportion of photoreceptor cells containing a single chromocenter was detected at the individual level, but not at the population level, as Fig. 2D shows that the left and right eyes have similar proportions of single to multiple chromocenters in the photoreceptor cells. Consistent with this result, *S. lessoniana* showed a dominant eye to a visual target; however, the side of the dominant eye was not determined at the population level [12].

### Light passing through the retina was reflected by the argentea

The orientation of photoreceptor cells in squid differs from that in vertebrates; light appears to reach the outer segment of each photoreceptor cell without interference from the chromocenters in the nucleus. We investigated whether chromocenters in squid could also affect light reception in photoreceptor cells. Therefore, we examined whether light passing through a lens could reach the inner surface of the argentea, the outer rim of the eye (Fig. 1B), and whether the inner surface of the argentea reflected light toward the outer segments. Currently, the role of the argentea is considered to be blocking light that does not pass through the lens by reflecting it [48, 49].

**Fig. 3:**
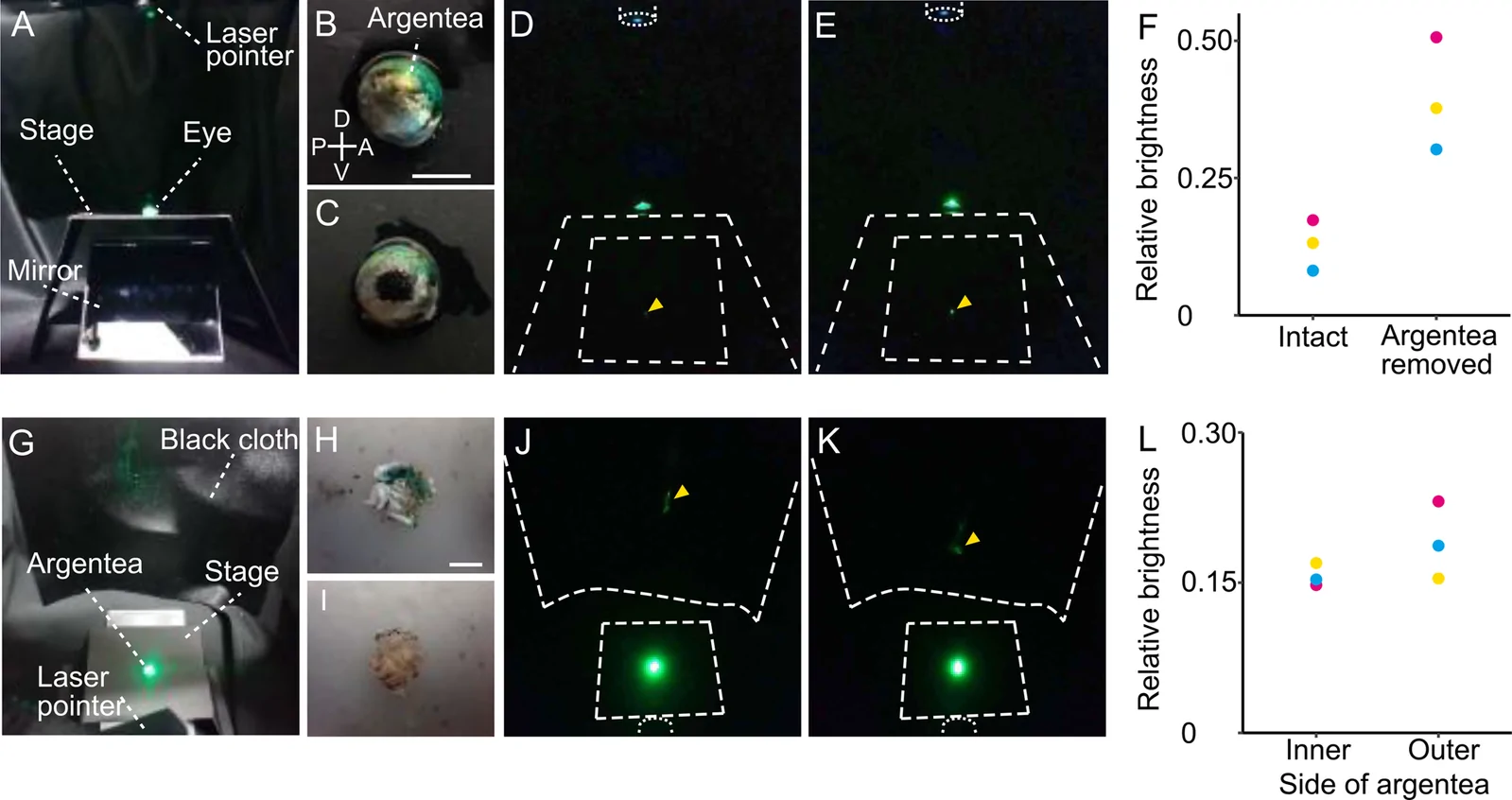
Light passing through the retina is reflected from the inner surface of the argentea. (**A**) Experimental setup to detect light passing through the eye. The eye was exposed to green laser light from a laser pointer. After passing through the eye and a hole in the stage beneath it, the light that reached a mirror was captured using a digital camera. This image was taken under light to illustrate the setup; however, experiments were performed in the dark. (**B**) Outer surface (argentea) of an intact eye. (**C**) Outer surface of an eye after partial excision of the argentea. (**D**) Light passing through the intact eye (arrowhead). (**E**) Light passing through the eye lacking part of the argentea (arrowhead). (**F**) Relative brightness of the light on the mirror after passing through the eye. (**G**) Experimental setup to detect light reflected from the argentea. The argentea was exposed to laser light. This image was taken under light to illustrate the setup; however, experiments were performed in the dark. (**H**) Outer surface of the argentea. (**I**) Inner surface of the argentea. (**J**) Light reflected from the outer surface of the argentea (arrowhead). (**K**) Light reflected from the inner surface of the argentea (arrowhead). (**L**) Relative brightness of the light reflected from the argentea. Each color in (**F, L**) represents the retina of a different animal. Scale bars: 5 mm (**B, H**)

First, we examined whether light reached the argentea after passing through the retina by emitting laser light (532 nm wavelength) toward the lens of the eye (Fig. 3A) with intact argentea (Fig. 3B) and with partially removed argentea (Fig. 3C). We observed light passing through the eye in both cases (Fig. 3D, E), indicating that light reached the argentea after passing through the retina and that light can pass through the argentea. The relative brightness of the light that passed through the eye with argentea was approximately one-third that of the light that passed through the eye without argentea (Fig. 3F). This result indicates that the argentea absorbs and/or reflects most of the light passing through the retina.

Next, we examined whether light was reflected from the inner surface of the argentea (Fig. 3G). We emitted laser light toward the outer and inner surfaces of the flattened argentea (Fig. 3H, I). As the outer surface of the argentea reflected light (Fig. 3J), we found that light was also reflected from the inner surface of the argentea (Fig. 3K). The relative brightness of the light reflected from the inner surface was comparable to that from the outer surface (Fig. 3L). These results indicate that the inner surface of the argentea reflects light toward the outer segments of the photoreceptor cells.

### Photoreceptor cells containing a single chromocenter could efficiently collect light on the outer segment

**Fig. 4:**
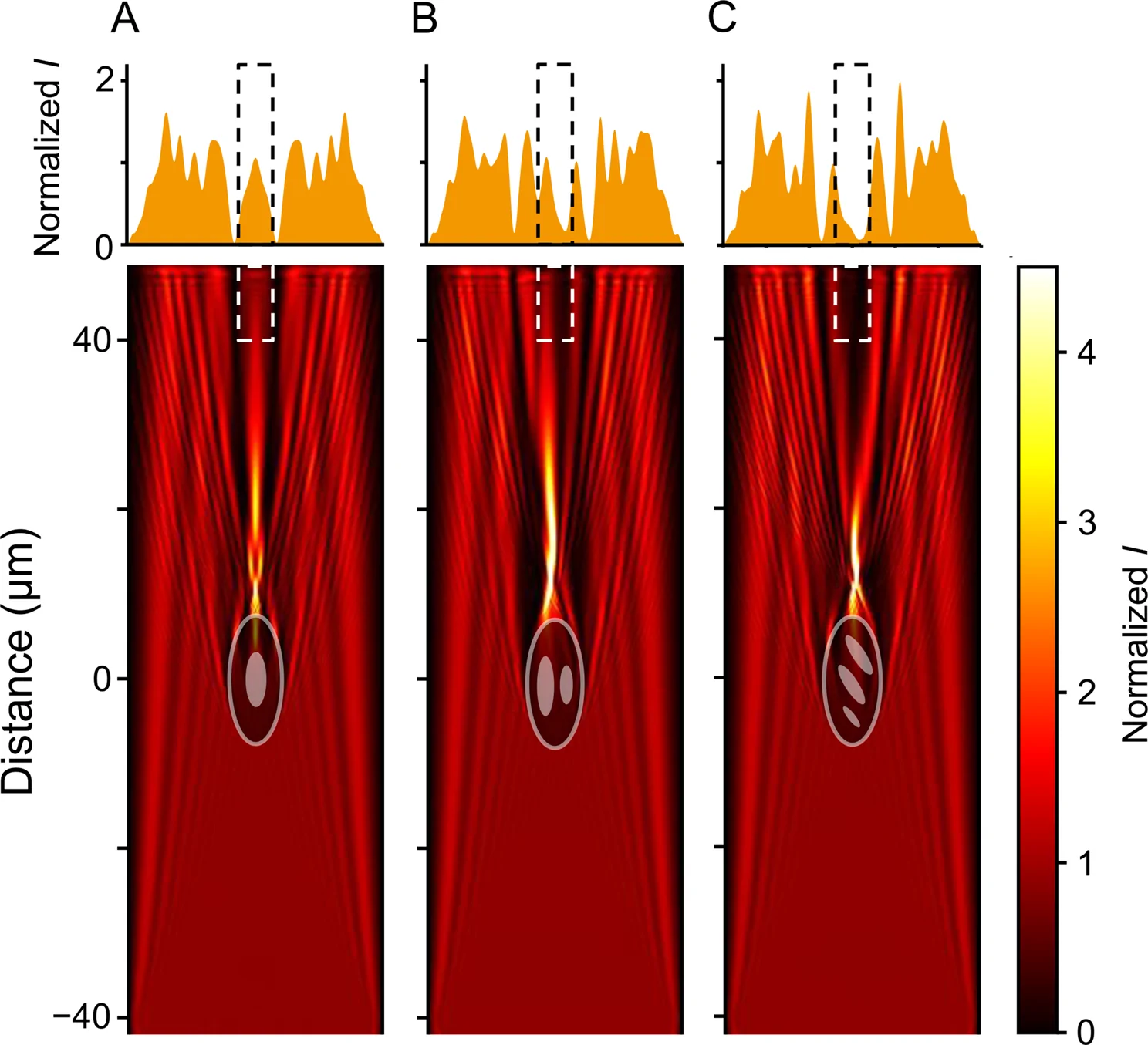
Simulation of light interference caused by heterochromatin structures in photoreceptor cells. The simulation analyzed how light reflected from the argentea (bottom of the heatmap) reached the outer segment (surrounded by white dashed lines) of photoreceptor cells containing a peripheral heterochromatin rim with one (**A**), two (**B**), or three (**C**) chromocenters. The heat maps represent normalized light intensities (normalized *I*; see Methods). The location of the outer segment of photoreceptor cells was determined from the distance between the top of the photoreceptor cell layer and the bottom of the outer segment layer. The graph (top) shows the mean normalized *I* in the outer segment layer between 40 and 49 μm from the center of the nucleus. The sizes of the peripheral heterochromatin rims were based on the mean lengths of the longitudinal, major, and minor axes of the heterochromatin rims, and the orientation of the chromocenters was set according to those shown in Fig. 2C. Organelles and the cell membrane were not included in the simulation to simplify the calculations

A previous simulation of mouse rod cells forming a single chromocenter showed that the chromocenter acts as a converging lens that focuses light on the outer segments of rod cells [32]. We next investigated whether the heterochromatin structure of photoreceptor cells in squid could also act as a lens and help collect light reflected from the argentea onto the outer segment by performing a 3D-FDTD simulation [44]. The simulation compared the light intensities at the outer segments of photoreceptor cells containing one to three chromocenters. Because the outer segments are tightly packed within the retina [3], we regarded the outer segment as forming a rectangular prism containing a square base and estimated the length of one side of the square by calculating the square root of the posterior area divided by the number of photoreceptor cells contained in the area (4.20 μm on average). The distance between the outer segment and the heterochromatin rim was set to that of the photoreceptor cell whose nucleus is located at the inner surface of the photoreceptor cell layer (40 μm on average). The simulation revealed that the light intensity at the outer segment was stronger in photoreceptor cells containing a single chromocenter within the peripheral heterochromatin rim than in those containing multiple chromocenters (Fig. 4). This result suggests that light reflected from the argentea is more efficiently collected on the outer segment of photoreceptor cells containing a single chromocenter, suggesting that the dominant eye, which has a larger proportion of photoreceptor cells containing a single chromocenter, has better contrast sensitivity than the non-dominant eye.

## Discussion

This study revealed that lateralization of the retinal structure was correlated with visual lateralization toward conspecifics in *S. lessoniana*. Lateralization of photoreceptor cell density and focal length was not significantly correlated with visual lateralization, whereas the proportion of photoreceptor cells containing a single chromocenter was. Although small sample sizes in this study (*n* = 5) may affect the robustness of this conclusion, these results suggest that an animal preferentially faces the left or right eye, which has a larger proportion of photoreceptor cells containing a single chromocenter, toward conspecifics. In squid, the orientation of photoreceptor cells differs from that in vertebrates; light appears to reach the outer segment of each photoreceptor cell without interference from the chromocenters in the nucleus. This raised the possibility that chromocenters may influence light reception via mechanisms distinct from those in vertebrates [32]. We indeed found that light was reflected from the inner surface of the argentea, indicating that the reflected light and light passing through the lens from outside the eye were collected together on the outer segment of the photoreceptor cells. Our model suggests that the outer segment can more efficiently collect reflected light in photoreceptor cells containing a single chromocenter. These results suggest that the photoreceptor cells in the dominant eye collect more light on the outer segment, such that the dominant eye achieves better contrast sensitivity than the non-dominant eye.

Body patterns and behavioral actions represent visual signals from conspecifics in *S. lessoniana* [1]. As behavioral signals such as movement in the water column, prey capture, and predator avoidance [50, 51] do not require high contrast sensitivity, visual lateralization may facilitate intraspecific communication mediated by such weak visual signals that are produced by iridophores, leucophores, or chromatophores [48, 49, 52]. The presence of a single chromocenter in rod cells helps collect light as a lens and increases contrast sensitivity in the mouse visual system, which would aid nocturnal life [32, 33]. This study revealed that visual lateralization toward conspecifics was correlated with lateralization in the proportion of photoreceptor cells containing a single chromocenter at the juvenile stage, when schooling behavior is observed during day and night. Particularly at night, *S. lessoniana* may use light reflected by the argentea to increase contrast sensitivity or enhance weak light signals through the chromocenters to maintain the schooling behavior [50, 51, 53, 54]. Rod cells of nocturnal mammalian species generally contain a single chromocenter, whereas those of diurnal mammals possess multiple chromocenters [32]. Investigating whether the relationship between lifestyle and heterochromatin structures in photoreceptor cells is also applicable to invertebrates and other vertebrate species would be of notable scientific interest.

Lateralization at the level of sensory organs has also been reported in various animal species. For example, in European starling, *Sturnus vulgaris*, long- and medium-wavelength-sensitive single cones are more abundant in the left eye, whereas double cones are more abundant in the right eye [55]. In honeybee, *Apis mellifera*, the putative olfactory sensilla were more abundant on the right antenna surface [56]. These lateralizations have been suggested to be the cause of behavioral lateralization in color discrimination and movement detection in *Sturnus vulgaris* and short-term recall of olfactory memories in *A. mellifera*. As the sensory input is different between the left and right sensory organs in these species, sensory processing in the brain would also be lateralized. Indeed, in vivo calcium imaging further showed that odor-evoked activity patterns are more distinct in the right antennal lobe in *A. mellifera* [57]. Our study revealed that the lateralization of the proportion of photoreceptor cells containing a single chromocenter is correlated with visual lateralization in *S. lessoniana*. Further electrophysiological studies are also required to clarify the contribution of this retinal lateralization to sensory processing in the brain and to visual lateralization toward conspecifics, in order to elucidate the mechanisms underlying visual lateralization in intraspecific communication in *S. lessoniana*.

In *S. lessoniana*, heterochromatin in the nuclei of photoreceptor cells is located both in the peripheral rim and inside the rim as a single or a few chromocenters. To our knowledge, heterochromatin in vertebrates and gastropods forms either a single chromocenter without the peripheral rim or multiple chromocenters within the heterochromatin peripheral rim [58]. In other reported cephalopod species, heterochromatin in photoreceptor cells forms multiple chromocenters and a peripheral rim [59–62]. Thus, this study is the first report of an animal species that has a single chromocenter surrounded by a heterochromatin rim in photoreceptor cells.

Photoreceptor cells in *S. lessoniana* have one to five chromocenters in their nuclei. Because the chromocenter is generally considered a heterochromatin structure [32], each photoreceptor cell may exhibit a different gene expression pattern depending on the number of chromocenters formed in the nucleus. This may indicate that photoreceptor cells and photoreception in squid retinae are more heterogeneous than expected, although only a single opsin gene exists in the genome of *S. lessoniana* [46]. To understand the convergent evolution of lens eyes between cephalopods and vertebrates, further genetic studies are required to investigate how differences in heterochromatin distribution contribute to gene expression patterns and cellular functions across these lineages.

Currently, the argentea is thought to prevent light that does not pass through the lens from entering the eye by reflecting it [48, 49]. The tapetum, a reflective layer in the fish eye, reflects light that passes through the retina, thereby enhancing light absorption on the outer segment under low-light conditions [63]. Although the inner surface of the argentea may not reflect light as efficiently as the tapetum, the argentea may serve a function similar to that of the tapetum, reflecting light toward photoreceptor cells to enhance light absorption on the outer segment in dim environments [64]. This similarity may imply convergent evolution of the outer rim of the lens eyes between cephalopods and vertebrates to optimize visual sensitivity in dim environments.

## Conclusions

This study revealed that lateralization in the proportion of photoreceptor cells containing a single chromocenter was positively correlated with visual lateralization toward conspecifics in *S. lessoniana*, whereas neither photoreceptor cell density nor focal length showed a significant correlation. We also demonstrated that light passing through the retina is reflected from the inner surface of the argentea, and simulations indicated that the light was efficiently collected on the outer segment of photoreceptor cells containing a single chromocenter. These findings suggest that lateralization of heterochromatin structure within the nuclei of photoreceptor cells may contribute to intraspecific communication by enhancing weak visual signals produced by chromatophores, leucophores, or iridophores of conspecifics. Further studies are required to investigate whether lateralization of retinal structure directly contributes to visual lateralization.

## Electronic supplementary material

Below is the link to the electronic supplementary material.

Supplementary material 1

Supplementary material 2

Supplementary material 3

Supplementary material 4

## Data Availability

The datasets and the code of the FDTD simulations supporting the conclusions of this article are included within the additional files.
