# Supplementary material for: Lateralization of retinal structure correlated with behavioral lateralization in the oval squid Sepioteuthis lessoniana
Source: Zoological Lett. 2026 Sep 17;12:15. doi: 10.1186/s40851-026-00268-5 (PMC13595632; doi:10.1186/s40851-026-00268-5)
Supplement: Supplementary file 1 [file 40851_2026_268_MOESM1_ESM.pdf]

One chromocenter

July 9, 2026

[ ]:
```python
import meep as mp
import numpy as np
import matplotlib.pyplot as plt

resolution = 33
cell_size  = mp.Vector3(30, 100, 30)
pml_layers = [mp.PML(1)]

nucleus_size = mp.Vector3(6.8, 16.1, 6.0)
heterochromatin_index = 1.415
euchromatin_index     = 1.385
surrounding_index     = 1.360

wavelength = 0.5
frequency  = 1 / wavelength
period     = wavelength

geometry = [
    mp.Ellipsoid(size=nucleus_size,
                 material=mp.Medium(index=heterochromatin_index)),
    mp.Ellipsoid(size=nucleus_size*3/4,
                 material=mp.Medium(index=euchromatin_index)),
    mp.Ellipsoid(size=nucleus_size*1/3,
                 material=mp.Medium(index=heterochromatin_index))
]

symmetries = [mp.Mirror(mp.Z, 1)]

sources = [
    mp.Source(
        src=mp.ContinuousSource(frequency=frequency),
        component=mp.Ez,
        center=mp.Vector3(0, -40),
        size=mp.Vector3(cell_size.x, 0,cell_size.z)
    )
]
```

```python
sim = mp.Simulation(
    cell_size=cell_size,
    geometry=geometry,
    sources=sources,
    boundary_layers=pml_layers,
    resolution=resolution,
    symmetries=symmetries,
    default_material=mp.Medium(index=surrounding_index),
    Courant=0.5
)

nx = int(cell_size.x * resolution)
ny = int(cell_size.y * resolution)

x = np.linspace(-cell_size.x/2, cell_size.x/2, nx)
y = np.linspace(-cell_size.y/2, cell_size.y/2, ny)

ix = np.where((x >= -15) & (x <= 15))[0]
iy = np.where((y >= 40) & (y <= 49))[0]

region_center = mp.Vector3()
region_size   = mp.Vector3(cell_size.x, cell_size.y, 0)

ref_point = mp.Vector3(0, -40)

full_buf    = np.zeros((nx, ny))
partial_buf = np.zeros((len(ix), len(iy)))
ref_buf     = 0.0

sample_count = 0

def record_fields(sim):
    global full_buf, partial_buf, ref_buf, sample_count

    ez = sim.get_array(
        center=region_center,
        size=region_size,
        component=mp.Ez
    )
    ez2 = np.abs(ez)**2

    full_buf    += ez2
    partial_buf += ez2[np.ix_(ix, iy)]

    ez_ref = sim.get_field_point(mp.Ez, ref_point)
    ref_buf += np.abs(ez_ref)**2
```

```
    sample_count += 1

sim.run(until=250 * period)
sim.run(mp.at_beginning(record_fields))
sim.run(mp.at_every(sim.fields.dt, record_fields), until=period)

full_avg    = full_buf    / sample_count
partial_avg = partial_buf / sample_count
ref_avg     = ref_buf     / sample_count

partial_norm = partial_avg / ref_avg
full_norm    = full_avg    / ref_avg

out = np.zeros((len(iy)+1, len(ix)+1))
out[0,1:] = x[ix]
out[1:,0] = y[iy]
out[1:,1:] = partial_norm.T

np.savetxt(
    "meep_normEz2_x-15to15_y40to49_CC1.csv",
    out, delimiter=",", fmt="%.6e"
)

plt.figure(dpi=300)

im = plt.imshow(
    full_norm.T,
    origin="lower",
    extent=[-cell_size.x/2, cell_size.x/2,
            -cell_size.y/2, cell_size.y/2],
    cmap="hot",
    vmin=0,
    vmax=4.5
)

cbar = plt.colorbar(im)
cbar.set_label(" Ez²  /  Ez² ref")

cbar.set_ticks(np.arange(0, 5, 1))

plt.xlabel("X (μm)")
plt.ylabel("Y (μm)")
plt.title("Normalized  Ez²  (steady-state, 1-period average)")

plt.savefig("meep_normEz2_full_CC1.svg")
plt.close()
```
